# Brute force prey metabarcoding to explore the diets of small invertebrates

**DOI:** 10.1002/ece3.11369

**Published:** 2024-05-06

**Authors:** Snorre Flo, Anna Vader, Kim Præbel

**Affiliations:** ^1^ Department of Arctic Biology The University Centre in Svalbard Longyearbyen, Svalbard Norway; ^2^ Faculty of Biosciences, Fisheries and Economics UiT The Arctic University of Norway Tromsø Norway; ^3^ The Norwegian College of Fishery Science (NFH) UiT The Arctic University of Norway Tromsø Norway; ^4^ Department of Forestry and Wildlife Management Inland Norway University of Applied Sciences Elverum Norway

**Keywords:** blocking primers, prey metabarcoding, sequence data, species interactions

## Abstract

Prey metabarcoding has become a popular tool in molecular ecology for resolving trophic interactions at high resolution, from various sample types and animals. To date, most predator–prey studies of small‐sized animals (<1 mm) have met the problem of overabundant predator DNA in dietary samples by adding blocking primers/peptide nucleic acids. These primers aim to limit the PCR amplification and detection of the predator DNA but may introduce bias to the prey composition identified by interacting with sequences that are similar to those of the predator. Here we demonstrate the use of an alternative method to explore the prey of small marine copepods using whole‐body DNA extracts and deep, brute force metabarcoding of an 18S rDNA fragment. After processing and curating raw data from two sequencing runs of varying depths (0.4 and 5.4 billion raw reads), we isolated 1.3 and 52.2 million prey reads, with average depths of ~15,900 and ~120,000 prey reads per copepod individual, respectively. While data from both sequencing runs were sufficient to distinguish dietary compositions from disparate seasons, locations, and copepod species, greater sequencing depth led to better separation of clusters. As computation and sequencing are becoming ever more powerful and affordable, we expect the brute force approach to become a general standard for prey metabarcoding, as it offers a simple and affordable solution to consumers that is impractical to dissect or unknown to science.

## Graphical abstract

We have used deep brute force metabarcoding to characterize prey interactions of invertebrates. Not requiring a priori sequence information or dissection, the brute force approach may enable studies on many small and enigmatic invertebrates.

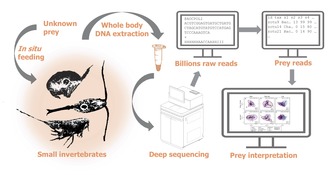

## 1 INTRODUCTION

Understanding ecosystems requires the construction and modeling of complex networks that represent various species interactions, and abiotic factors. In such ecosystems, small invertebrates perform important tasks as consumers, prey, decomposers, and pollinators, and are thus critical to include (Kellert, 1993), but large and charismatic animals like birds and mammals have to date garnered the bulk of trophic ecologists' attention (Eisenhauer & Hines, 2021). Traditionally, dietary studies have been conducted through direct observation of feeding behavior, or morphological identification of prey species in regurgitate, stomach, or fecal contents (Pompanon et al., 2012; Sousa et al., 2019; Symondson, 2002), but these approaches are highly impractical for a large portion of invertebrates whose small bodies complicate stomach content extraction and produce comparatively small fecalia.

Microscopy has helped identify small prey from small consumers, but is laborious and biased in favor of big, well‐preserved prey (Berg, 1979), and demands morphological and taxonomic expertise (Pompanon et al., 2012). Conversely, experimental monitoring of communities over time can detect trophic interactions, and even allow quantifying ingestion rates, but struggles in reproducing the natural variability. The species assortment would typically be limited to prey expected in advance, or that co‐occurred with the consumer if a natural sample was used as the starting point. Prey metabarcoding has become popular because it allows identification of diverse prey from complex and partly digested material, and does not require considerable a priori knowledge of prey, or taxonomical or morphological knowledge from the researcher (Casper et al., 2007). In broad strokes, metabarcoding includes extraction of DNA from dietary material—most often regurgitate, feces or stomach content, PCR amplification of target DNA (the marker gene or barcode), sequencing of PCR amplicons, and culminates with taxonomic identification by comparing obtained sequences to those in a reference database (Santoferrara, 2019).

Deciding on a dietary material is an important step that depends on the logistics and ethics of sampling, or the nature of the species being studied, such as its size or tendency for violence (Pompanon et al., 2012). Medium‐sized crustaceans like northern shrimp (*Pandalus borealis*) may be suitable for excision of stomach content (Urban et al., 2022), but small invertebrates (<1 mm) are challenging to dissect, and may require whole body extraction (e.g., Novotny et al., 2021; Zamora‐Terol et al., 2020). This comes at a cost, however, because the majority of DNA in the sample will naturally stem from the consumer itself (Piñol et al., 2014, 2015). An overabundance of consumer DNA may also be a challenge when other materials (e.g., feces or gut content) are sampled (Kohn & Wayne, 1997), but its concentration becomes severely exalted in extracts of whole bodies (Piñol et al., 2014). Hence, the DNA of interest is in minority, while the unexciting consumer DNA will compose a competitive majority.

Conceptually different approaches have been developed to enable prey studies from such “mixed” DNA samples, for instance reducing amplification of consumer DNA by using blocking primers. Nowadays most popular approach was spurred when Nielsen et al. (1991) researched synthetic analogs to DNA. A polymer with peptide instead of a sugar‐phosphate backbone showed particular promise because it formed stable hybrid duplexes with DNA (Nielsen et al., 1991). So‐called peptide nucleic acids (PNAs) had higher melting temperatures than DNA (Egholm et al., 1993), went unrecognized by DNA polymerases, and could not initiate amplification by PCR (Orum et al., 1993). PNAs could be introduced prior to PCR to hybridize irreversibly with a target sequence and thereby suppress its amplification (Orum et al., 1993). Comparatively rare but interesting sequences would thus be allowed to replicate to detectable abundances (e.g., eukaryote parasites of blue crab, Troedsson et al., 2008). Other variants of blocking primers have also been put to the test, such as oligonucleotides modified with inhibitory C3 spacers (Deagle et al., 2009; Vestheim & Jarman, 2008). Since then, blocking primers have been used to study the prey of many different animals and with sample material ranging from whole‐body extracts of copepods (Cleary et al., 2016, 2017; Durbin & Casas, 2014; Novotny et al., 2021; Ray et al., 2016; Zamora‐Terol et al., 2020), to dragonflies and apex canine predators (Morrill et al., 2021; Shi et al., 2021).

Although blocking primers have enabled many prey studies, there are issues that warrant attention. Like universal PCR primers typically used in metabarcoding, blocking primers can introduce bias during amplification (Elbrecht & Leese, 2015; Leray & Knowlton, 2017; Piñol et al., 2015). With universal primers, primer‐template mismatches and stochasticity may result in skewed relative abundances of species of interest (Sipos et al., 2007). Blocking primer bias also relates to primer‐template mismatches, but it is rather the lack of them that leads to unreliable results. A blocking primer should have zero mismatches with the consumer, and as many as possible mismatches with prey to limit off‐target interactions. Piñol et al. (2015) showed that a blocking primer with four and five mismatches to interesting prey decreased their relative abundances. Hence, PNAs and blocking oligonucleotides may introduce strong taxonomic biases during amplification (Piñol et al., 2015, 2019). Furthermore, the production of specific and ultimately successful blocking primers remains an expensive chemical procedure and relies on both consumer and prey sequences before the design can begin.

We have explored a simple, cost‐efficient, and versatile approach to explore eukaryote prey of small invertebrate consumers. By sequencing deep to offset the overabundance of consumer DNA, we show that one can obtain ample 18S rDNA prey reads from mixed whole‐body copepod extracts, while at the same time avoiding the costs and laborious design of potentially biased blocking primers. Conceptually, we argue that the brute force method holds a lot of promise because current sequencing and computation enable the acquiring and processing of large amounts of data, and continuous development will only improve these capabilities (Lightbody et al., 2019). Through two sequencing runs (a pilot and a full‐scale) using two commercially available NGS platforms (Illumina HiSeq4000 and the scalable Illumina NovaSeq6000), we tested the brute force approach for the first time with marine invertebrates. Specifically, we tested if the increased sequencing throughput of the Illumina NovaSeq6000 platform was useful for offsetting the expected overabundance, and hence for identification of prey interactions. We report on the effect of sequencing depth for resolving prey composition and discuss the advantages and caveats of the brute force methodology for prey studies of small consumers.

## 2 MATERIALS AND METHODS

### 2.1 Sample collection

Copepods were collected on four seasonal cruises from the central Barents Sea to the Arctic Nansen basin northeast of Svalbard, Norway (Table S2). Cruises occurred during Autumn (5–27 August 2019), early winter (28 November–17 December 2019), late winter (2–24 March 2021), and early spring (27 April – 20 May 2021), and visited three stations on the Barents Sea central shelf (76.00 N, 31.22 E), northern shelf (79.72 N, 34.32 E), and Nansen basin (81.83–82.16 N, 28.15–29.84 E, positions varied due to sea‐ice drift, Figure S1). Small‐sized mesozooplankton (<1 mm) were collected in vertical 64 μm Bongo‐net hauls (to full depth or max. 1000 m, ascent = 0.3 m s^−1^, descent = 0.5 m s^−1^, 60 cm mouth diameter). All large and/or gelatinous animals (1–10 cm) were removed, and the remaining suspension was sieved (64 μm) to discard seawater. Ice‐cold ethanol (96%, −20°C) was then used to rinse retained mesozooplankton, before transfer into a sample bottle. The container was topped up with ice‐cold ethanol and stored at −20°C.

*Microsetella norvegica* (Boeck, 1865), *Microcalanus* spp. (*M. pygmaeus* or *M. pusillus*, Sars G. O., 1900–1903), and *Oithona similis* (Claus, 1866) were morphologically identified under a stereomicroscope (Table S2). Up to 14 individuals per species and station were picked where available. Each specimen was thoroughly rinsed individually three times in Milli‐Q water, and transferred to tissue lysis buffer (E.Z.N.A Tissue DNA kit, Omega Bio‐tek). Surface sterilization with bleaching was excluded since the minute body size (<1 mm) of the copepods analyzed herein raised concerns regarding how the treatment could penetrate and potentially alter the dietary signal. Also, existing literature, mostly based on arthropods, is in disagreement regarding the efficacy of bleaching, with one study indicating little effect on the overall dietary signal (Miller‐ter Kuile et al., 2021). DNA extraction was performed per manufacturer's protocol (“Tissue Spin Protocol,” E.Z.N.A® Tissue DNA kit, Omega Bio‐Tek), with a lowered elution volume of 2 × 50 μL elution buffer. One negative without material was included with every round of extraction.

We verified amplification in all copepods and lack of amplification in negative DNA extracts by PCR amplification using universal eukaryotic primers. The success of the amplifications was inspected by 1% agarose gel electrophoresis, confirming single‐band PCR products in all samples and no amplification in all extraction negatives.

### 2.2 Library preparation and sequencing

For sequencing, we amplified a short hypervariable fragment of the 18S SSU rRNA V7 region (~100–110 bp) with 18S_allshorts primers (Forward 5′‐TTTGTCTGSTTAATTSCG‐3′, and Reverse 5′‐GCAATAACAGGTCTGTG‐3′) (Guardiola et al., 2015). Broad coverage within Eukarya was verified with the Arb Silva TestPrime function (Klindworth et al., 2013). To enable the pooling of samples after amplification, primers were pre‐tagged with an 8‐base oligonucleotide, wherein at least three nucleotides differed between tags. The primers also contained a leading 5′‐end variable number of degenerate nucleotides (N, 2 ≤ 4) to increase sequence variability and hence Illumina sequencing quality (Wangensteen et al., 2018). PCR amplification and all downstream processing from here and onwards were conducted twice. First as a pilot with a limited number of samples and negatives (N_1_ = 79), and finally with a full set of samples and negatives (N_2_ = 456). Samples were processed using the exact same protocol unless stated otherwise.

PCR amplifications were performed in 20 μL reactions with 10.00 μL AmpliTaq Gold™ Master Mix (Applied Biosystems), 0.16 μL Bovine Serum Albumin (BSA, 20 μg μL^−1^), 5.84 μL ultrapure MQ water, 2.00 μL of 18S_allshorts Forward and Reverse primer mix (2.5 μM each), and 2.00 μL DNA template. Thermal cycling consisted of an initial denaturation step (10 min, 95°C), and 35 cycles of denaturation (30 s, 95°C), annealing (30 s, 45°C), and elongation (30 s, 72°C). For each PCR plate, a subsample of real samples (*n* = 9) and a PCR negative were tested on a 1% agarose gel to verify amplification and a lack thereof, respectively. PCR amplicons were then pooled, purified with MinElute™ spin‐columns (Qiagen, Hilden, Germany), and quantified using the broad‐range dsDNA assay on a Qubit 4™ fluorometer (Invitrogen by ThermoFisher). Sequencing‐ready libraries were prepared from purified pools in accordance with the NEXTflex™ PCR‐Free DNA Sequencing Kit (Bioo Scientific, Austin, TX, USA). Here, DNA templates (3000 ng total for each library) were first purified by size (retaining fragments >150 bp) with magnetic Agencourt AMPure XP beads (Beckman Coulter Genomics, CA, USA), then adenylated, and ligated with Illumina‐compatible adapters. We used several Illumina‐compatible adapters (NEXTflex™ DNA Barcode Adapter; Bioo Scientific) to distinguish libraries of 96 samples. The libraries were quantified by qPCR using the NEBNext® Library Quant Kit for Illumina® (New England Biolabs, MA, USA) to verify successful preparation. For the pilot sequencing one library of 79 samples (75 real and 4 extraction negatives), was sequenced using 150 Paired‐End (PE) chemistry on a HiSeq 4000 platform (Novogene Co., Ltd.). For the full sequencing run, five libraries with a total of 456 samples (437 real and 19 extraction negatives), were sequenced using 150 PE chemistry on two lanes of a NovaSeq6000 platform (Novogene Co., Ltd.).

### 2.3 Bioinformatic processing

A custom bioinformatics pipeline based on the OBITools (v. 1.2.12, (Boyer et al., 2016)) and VSEARCH (v. 2.9.1, (Rognes et al., 2016)) software suites and the unoise3 algorithm (Edgar, 2016) was developed to process reads (available at: https://github.com/snflo/bruteforce). Forward and reverse reads were paired with the illuminapairedend function. The reads were then passed via criteria selecting aligned reads of high quality (score > 40.00), assigned to sample, and trimmed based on the sequences of the primers and attached oligonucleotide tags (ngsfilter). Reads were further selected for sequence non‐ambiguity and read lengths between 80 and 120 bp. To enable faster processing, the data were split by sample, and distributed over several CPUs whom in parallel performed dereplication (obiuniq), sorting (vsearch ‐‐sortbysize), denoising with removal of sequences with less than four reads (vsearch ‐‐cluster_unoise, −‐minsize 4, −‐unoise_alpha 8) and chimera removal (vsearch ‐‐uchime3_denovo). Resulting sequence variants are hereon referred to as zero‐radius operational taxonomic units (zOTUs). After the most computationally heavy processing, all sample subfiles were concatenated, and zOTUs were reassigned to sample (obiuniq). Finally, taxonomy was assigned to the Protist Ribosomal database (PR2, v.4.14.0, Guillou et al., 2013) using blastn (BLAST+, v. 2.8.1, Altschul et al., 1990; Camacho et al., 2009).

### 2.4 Curation of prey

To obtain datasets with putative prey only, the assigned reads were subjected to a two‐step curation process in R studio (v. 4.1.3). First, the reads were manually filtered based on PR2 assigned taxonomy using “tidyverse” functions (Wickham et al., 2019). All Maxillopoda (which include both currently accepted taxonomic groups Oligostraca and Multicrustacea) reads were discarded to remove consumer DNA. We acknowledge that maxillopods may compose a food source for the species studied but the short read‐length used to capture prey from partly digested materials did not allow for distinguishing DNA from maxillopod prey and consumer. Taxa known to interact with copepods (any Copepoda) in symbiosis (parasitism, commensalism, and mutualism) were recorded from current literature (Bass et al., 2021; Cleary et al., 2017; Cleary & Durbin, 2016; Zamora‐Terol et al., 2021) and used to discard likely non‐dietary interactions. By inspection, we discarded several zOTUs assigned to unlikely prey including seed plants, insects, and mammals. Putative contaminants from the marine environment were likewise discarded, notably large gelatinous organisms (Cnidaria, Ctenophora). We acknowledge that also these taxa may have a dietary origin, but we consider it more plausible that most of the sequences originated from the batch sample from which the copepods were picked. Gelatinous organisms are sticky and fragile and have been suspected of contaminating other studies of copepods using similar methodologies (Cleary et al., 2017). Decontam was used to identify and discard remaining contaminants (Davis et al., 2018). We chose the prevalence method, which compares the prevalence (presence/absence‐based frequency) of zOTUs in real samples (copepods) and extraction negatives to flag zOTUs that are likely contaminants. Relatively few contaminants (2 and 38 for pilot and full datasets, respectively) were identified and discarded at this stage. The remaining zOTUs were considered putative prey and were stored with metadata as phyloseq objects (McMurdie & Holmes, 2013).

### 2.5 Sample metrics and analyses

Read metrics (counts and number of zOTUs) were acquired using different summarizing functions during bioinformatic processing (grep, obigrep, gawk), and selection functions (tidyverse, Wickham et al., 2019) during taxonomic filtration in R. To isolate the effect of differential sequencing, we generated an additional dataset (referred to as “pilot full”) by sub‐setting—from the full dataset—the same real samples that were sequenced in the pilot (*n* = 75). To test how well the different datasets represent prey diversity, we performed rarefaction analyses with the rarecurve function (step = 10) of vegan (Oksanen et al., 2019), and calculated the average number of reads required to discover new zOTUs (rareslope, sample = sample total reads).

### 2.6 Testing the effect of sequencing depth on prey composition

Non‐metric multidimensional scaling (NMDS) plots were used to visualize how depth of sequencing and data transformation may influence prey composition resolution. Multiple variants were made to explore beta‐diversity using both occurrence and presence/absence‐based dissimilarity metrics. All ordinations were computed from sample‐wise dissimilarities from compositional data at zOTU‐level. The relative abundances of each zOTU were computed per sample by dividing the number of reads of each zOTU by the total of sample prey reads. Relative abundances were then used to compute Bray–Curtis dissimilarities (Bray & Curtis, 1957). Since a presence/absence‐based metric is less sensitive to biases introduced during PCR (e.g., preferential amplification), we chose to also generate Jaccard dissimilarities (Jaccard, 1901) from a presence/absence‐table wherein a 0.01% relative abundance threshold was enforced to denote presence or absence of zOTUs. We used Scree‐plots to find the appropriate dimensions for a conservative acceptance threshold of ≤0.1 stress. Final NMDS plots were calculated iteratively (trymax = 100) with adequate dimensions (*k* = 4 or 5) using the metaMDS function of vegan (Oksanen et al., 2019). PERMANOVA (nperm = 10,000) analyses were used to test if three explanatory variables (season, station, and species) accounted for the observed variance in prey composition. We subsequently tested if the grouped samples had homogenous and comparable dispersions (*p* ≥ .05), or if compositional differences in prey could be due to heterogenous dispersion among groups (*p* < .05, Betadisper).

## 3 RESULTS

### 3.1 Detected prey

Identified prey zOTUs belonged to a broad range of eukaryote taxa including metazoans (e.g., Chaetognatha, Urochordata, Rotifera), fungi (Ascomycota, Basidiomycota), ciliates (e.g., Spirotrichea), dinoflagellates (Dinophyceae, Dinophyta_X), heterokonts (e.g., Bacillariophyta, Chrysophyceae, Labyrinthulea), and radiolarians (Acantharea). Mean relative abundances of prey are available in the Table S4, and prey compositions are presented in greater detail in an upcoming publication (S. Flo, C. Svensen, K. Præbel, B. A. Bluhm and A. Vader, unpublished data).

### 3.2 Sequencing metrics

The pilot sequencing (*N* = 79 including sequencing blanks) led to a total of 412 million paired‐end raw reads, which initial processing steps (pairing, filtering by length, quality and ambiguity, and demultiplexing) reduced to 342 million (“Pilot,” Table 1). Removal of chimeric, erroneous, and rare sequences further reduced the dataset, and after taxonomic identification 284 mill. reads (69% of raw) distributed over 49,697 zOTUs were isolated. Most of the reads that were subsequently filtered out were assigned to the consumer taxon Maxillopoda (97.8% of assigned reads), whereas reads identified as contaminants or symbionts accounted for 1.6% and 0.2%, respectively. The final dataset of putative prey counted 1.2 million reads (0.4% of the assigned reads) in 1500 zOTUs. Distributed over 75 real samples, the pilot averaged 16,000 prey reads per copepod consumer. Prey zOTUs were further divided among 175 species identifiers from 155 genera.

**TABLE 1:** Summary of read and zOTU abundances before and during bioinformatic processing (Step 1–4), according to sample type (real samples or extraction negatives), and according to taxonomic identity (consumer, symbiont, contamination, prey).

Step	Pilot (HiSeq 150 PE, *N* = 79)			Full (2 × Novaseq 150 PE, *N* = 456)		
	Reads	zOTUs		Reads	zOTUs	
1. Raw PE reads	412,449,403	–	–	5,436,416,402	–	–
2. Paired, filtered, demultiplexed	342,785,829	–	–	4,857,351,483	–	–
3. Denoised, without chimeras or singletons (<4)	284,213,421	49,723	–	4,268,556,612	130,677	–
4. Assigned to taxonomy	284,212,399	49,697	–	4,268,371,437	129,940	–

Sample type			POA			POA
Extraction negatives (*n*_p_ = 4, *n*_f_ = 19)	10,801,345	1986	3.80	8,269,055	5046	0.19
Real samples (*n*_p_ = 75, *n*_f_ = 437)	273,411,054	48,252	96.20	4,259,081,079	129,182	99.78

Identified taxa in real samples			POA^†^			POA^†^
Consumer	267,342,579	43,766	97.78	4,153,774,204	90,725	97.53
Symbionts	587,313	599	0.21	8,160,170	4266	0.19
Contaminants	4,291,765	2387	1.57	44,980,919	11,800	1.06
Prey	1,189,397	1500	0.44	52,165,786	22,391	1.22

*Note*: The total number of samples (*N*) was presented for both sequencing runs, and the number of extraction negatives and real samples are indicated in parentheses for the pilot (*n*_p_) and for the full sequencing (*n*_f_). Sample types and identified taxa are also presented with percentage‐wise contributions to the total of assigned reads (percentage of assigned; POA) or to assigned reads from real samples (POA^†^).

The final sequencing, with an increased number of samples (*N* = 456 including sequencing blanks), yielded 5.4 billion paired‐end raw reads (“Full”, Table 1). Of these, approximately 4.3 billion reads (79% of raw) in 130,000 zOTUs were subsequently assigned to taxonomy. After discarding zOTUs assigned to Maxillopoda (97.5% of assigned reads), contaminants (1.1%), and symbionts (0.2%), the putative prey counted 52.2 million reads in 22,391 zOTUs. The full dataset zOTUs were further divided among 559 species from 476 genera. Putative prey reads corresponded to 1.2% of the assigned reads, or 1.0% of the raw reads, and a mean depth of ~120,000 prey reads per copepod consumer. Compared to dividends from relevant literature using dissection or blocking primers, the average prey reads per sample of both sequencing runs were more than two times greater (Table 2).

**TABLE 2:** A summary of recent studies using prey metabarcoding to assess copepod trophic interactions.

Ref.	Copepod consumers	Dietary source	Assigned 18S reads	18S prey reads	Avg. prey reads per sample	Fragment (bp)	[Blocking primer] per [DNA]	Bias	Requirements	Suitable consumer characteristics
This study (pilot)	*O*. *similis*, *M*. *norvegica*, *Microcalanus* spp.	Whole bodies	284,212,399	1,189,397	16,000	18S V7 (~100–110)	No	PCR primers, CNV	Upscaled sequencing	Any invertebrate
This study (full)	*O*. *similis*, *M*. *norvegica*, *Microcalanus* spp.	Whole bodies	4,268,371,437	52,165,786	120,000	18S V7 (~100–110)	No	PCR primers, CNV	Upscaled sequencing	Any invertebrate
Hirai et al. (2018)	*Calanus sinicus*	Dissected guts	2,919,386	106,266^a^	2678	18S V9 (~130)	No	PCR primers, CNV	Dissection	Dissectible invertebrates
Yeh et al. (2020)	*Calanus finmarchicus*	Dissected foregut	NA	NA	NA	18S V4 (NA)	No	PCR primers, CNV	Dissection	Dissectible invertebrates
Cleary et al. (2017)	*Calanus glacialis*	Whole bodies	11,266,639^b^	638,231	7975	18S V7 (~250)	20 μM PNA per ~0.5 ng μL^−1^ DNA	PCR primers, CNV, blocking primers	PNA design and costs	Any invertebrate whose target sequence is known
Cleary et al. (2016)	*Pseudocalanus minutus*, *P. newmani*, *P. acuspes*	Whole bodies	NA	28,456	618	18S V7 (~250)	20 μM PNA per ~0.5 ng μL^−1^ DNA	PCR primers, CNV, blocking primers	PNA design and costs	Any invertebrate whose target sequence is known
Ho et al. (2017)	*Calanus sinicus*	Dissected anterior digestive tracts	2,183,773^c^	NA	NA	18S V4 (~300–350)	2 mM PNA per ~1.7 ng μL^−1^ DNA	PCR primers, CNV, blocking primers	PNA design and costs, dissection	Dissectible invertebrates whose target sequence is known
Novotny et al. (2021)	*Temora longicornis*, *Acartia* spp., *Pseudocalanus* spp., *Centropages hamatus*	Whole bodies	NA (>37 mill. 16S + 18S reads)	NA	NA	18S V4 (~400)	Yes, NA [PNA] per NA [DNA]	PCR primers, CNV, blocking primers	PNA design and costs	Any invertebrate whose target sequence is known
Zamora‐Terol et al. (2020)	*T. longicornis*, *Acartia* spp., *Pseudocalanus* spp., *C. hamatus*, *Eurytemora affinis*	Whole bodies	12,800,000	1,200,000	NA	18S V4 (~400)	Yes, NA [PNA] per NA [DNA]	PCR primers, CNV, blocking primers	PNA design and costs	Any invertebrate whose target sequence is known

*Note*: The list includes the consumers studied, the dietary source of DNA extractions, the total numbers of assigned 18S reads from all sources (e.g., consumer, prey, symbiont, and contaminant), and prey. In addition, we report the average prey reads per sample (calculated from reported sample size and total prey reads), the 18S fragment, and its putative length (bp). The use of blocking primers is listed with concentrations of blocking primer per concentration of template DNA. Putative methodological biases are listed, and we outline the conceptual requirements and suitable consumers for each approach.

Abbreviation: NA, not available.

^a^ Prey reads are calculated from the reported proportions of total reads.

^b^ Unknown if reads are assigned or raw.

^c^ Unknown if reads are prey or all assigned.

### 3.3 Rarefaction

**FIGURE 1:**
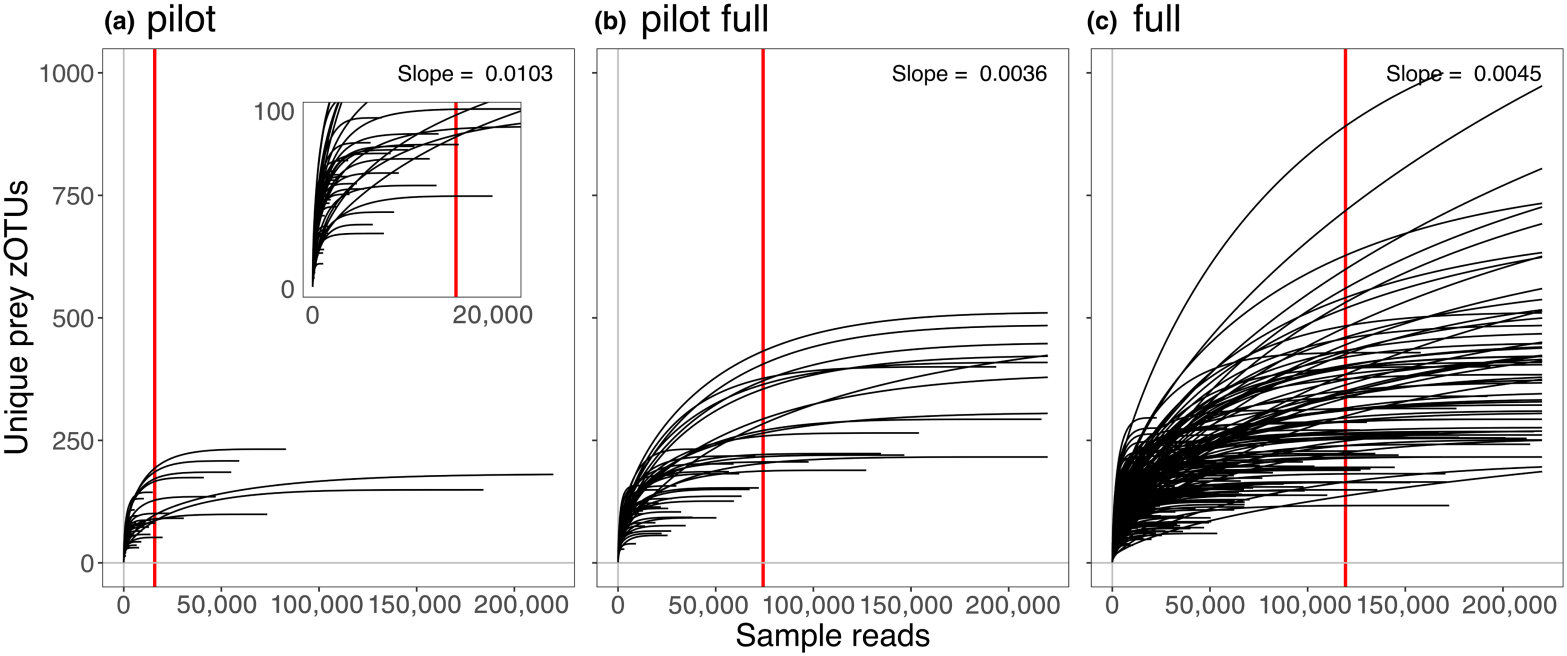
Rarefaction curves of prey data acquired from (a) the pilot (*n* = 75), (b) the upscaled sequencing run but with the copepod samples assessed in the pilot only (pilot full, *n* = 75), and (c) the full upscaled sequencing (*n* = 437). Vertical red lines indicate averages of (a) 16,000, (b) 74,000, and (c) 119,000 prey reads per copepod. Slopes indicate the average number of prey zOTUs discovered with each new read sequenced.

We used rarefaction to infer whether the sampling depths were sufficient to describe the full prey composition (i.e., zOTU richness). In theory, when rarefaction curves approximate a plateau, only a few novel zOTUs will be found with increased sequencing depth, thus indicating that the samples represent the full diversity of prey. The datasets all displayed plateau‐like curves (Figure 1), but curves of from the full sequencing run (pilot full, full) were arguably less steep. The average slope (i.e., zOTU discovery rate) of samples in the full dataset was smaller (0.0045, i.e., 4.5 new prey zOTUs per 1000 reads, Figure 1c) as opposed to 0.0103 for the pilot dataset (i.e., 10.3 new zOTUs per 1000 reads, Figure 1a).

### 3.4 Ordination and statistics

**FIGURE 2:**
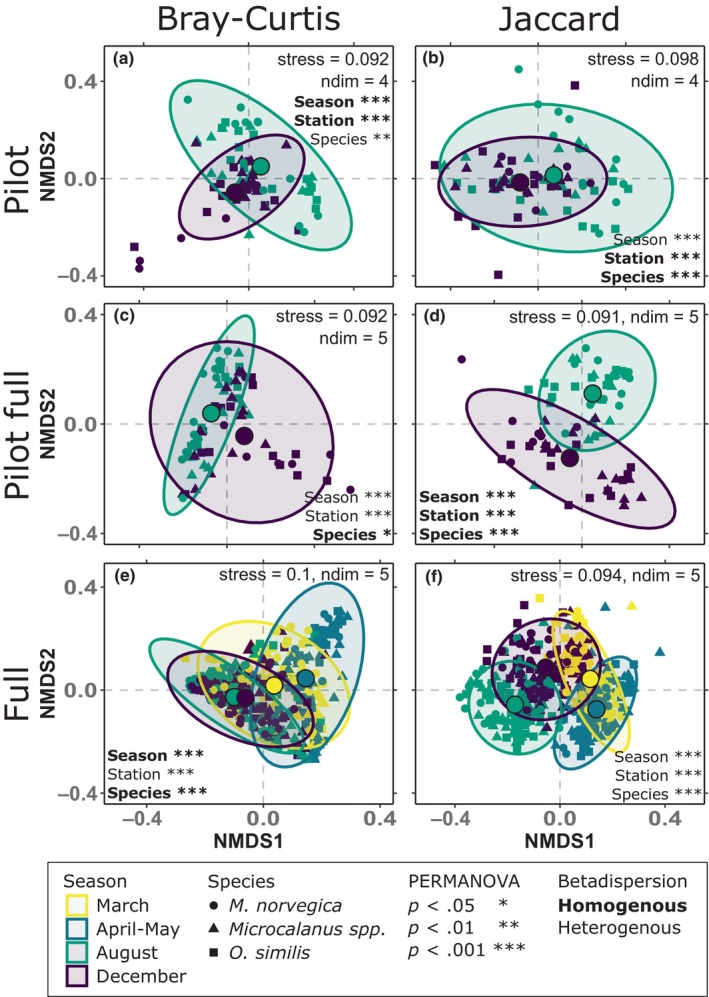
Nonmetric multidimensional scaling (NMDS) of dietary samples from (a, b) the pilot dataset (*n* = 75), (c, d) the same samples sequenced during the pilot, but from the full sequencing run (*n* = 75) and (e, f) the full sequencing with all samples (*n* = 437). Ordinations in (a, c, e) are based on Bray–Curtis dissimilarities, whereas (b, d, f) are based on Jaccard‐indexed dissimilarities computed from presence–absence data (zOTU presence = >0.01%). Colors distinguish the season of sampling, whereas shapes denote the identity of the consumer. Overlayed ellipses indicate 95% confidence levels and accompanying centroids show the average within‐group positions. Stress and number of dimensions (demanding a stress of ~0.1 or less) are shown. Significant differences between levels of groups (seasons, stations, and species) are shown based on PERMANOVAs, and in bold if the group dispersion was found to be homogenous (betadisper).

We prepared NMDS plots of all three datasets (“Pilot,” “Pilot full,” and “Full”, see explanation below) with abundance and presence/absence‐based dissimilarity metrics to investigate the importance of depth and transformation for determining the composition of prey (Figure 2). Depending on the dataset being used, ordination required four or five dimensions to reach a conservative and low stress level of 0.1. The pilot prey reads required fewer dimensions (*k* = 4) than the full dataset and the dataset consisting of the full subset (*k* = 5). Regardless of dataset or metric, prey composition differed significantly between copepods from different seasons and stations (PERMANOVA, *p* < .001, Figure 2). The most visually distinct clusters were found when using the season sampled for profiling prey compositions, and samples acquired during the pilot (“Pilot,” Figure 2a,b) formed less distinct clusters than those from the full sequencing. The same physical samples subset from the full dataset (“Pilot full,” Figure 2c,d) formed more divergent clusters. Ordination of the complete set of samples (“Full,” Figure 2e,f) returned a pattern typical of a seasonal transition, with prey compositions from successive seasons overlapping, and samples from disparate seasons (e.g., August and April/May) forming separate clusters. Successive Betadisper tests (Table S3) indicated however that the clusters observed may be influenced by heterogenous dispersion (e.g., Figure 2f). The copepod species sampled was a less significant predictor of pilot and pilot full diets (Figure 2a,c) when using Bray–Curtis as dissimilarity metric (*p* = .003 and *p* = .03, respectively), than with Jaccard. For both datasets with greater depth (“Pilot full” and “Full”), Jaccard dissimilarities led to visually greater separation of clusters.

## 4 DISCUSSION

The pragmatic approach applied here facilitated prey studies of small‐sized copepods despite an overabundance of noninformative consumer DNA. By scaling up the sequencing to offset losses in consumer reads, we avoided using expensive and potentially biased blocking primers. While the pilot sequencing resulted in approximating the full diversity of our samples, deeper sequencing reduced the zOTU discovery rate from 10.3 in the pilot to 3.6 and 4.5 new prey zOTUs per 1000 reads in the full pilot and full datasets, respectively. Our results from small‐sized marine copepods, which are known to consume a wide diversity of prey, thus suggest that upscaled brute force sequencing is a suitable general method for determining the prey of small consumers, regardless of a priori access to the consumer sequence or possibility of dissection.

### 4.1 Sequencing; how deep is deep enough?

The brute force concept was initially tested by Piñol et al. (2014) to determine the prey of the *Oedothorax fuscus* spider. Using a single pooled sample of 109 individual extracts they acquired what they called “ample” prey reads. Most sequence reads originated from the consumer, although the percentage of *O. fuscus* reads to the total was not reported. Their overall recovery was 61,000 prey reads, translating to 6% of all assigned reads. In our study, the prey recovery rates were much lower, with prey sequences accounting for only 0.44% and 1.22% of the assigned reads in the “pilot” and “full” sequencing runs, respectively. Thus, deeper sequencing was needed to acquire enough reads to cover the full diversity of prey species.

Nonetheless, if comparing prey read metrics with recent studies using PNA or other blocking primers (Table 2), it becomes clear that the applied brute force approach yielded a high absolute prey read output (1.2 and 52.2 mill. prey reads), regardless of high consumer losses. Reaching similar numbers with the use of blocking primers, a recent study of Baltic Sea copepods reported 1.2 million prey reads from an unknown number of samples (Zamora‐Terol et al., 2020). The use of a PNA blocker also did not eliminate the consumer DNA problem completely, given that the total read count before discarding Maxillopoda ASVs was 12.8 million reads. Hence, despite using blocking primers, 89% of the sequences were uninformative and discarded. Cleary et al. (2017) also acquired a large dataset with a total of 11.3 million reads from 80 samples of *Calanus glacialis* copepods. Although a PNA probe also here was used to block amplification of *Calanus* spp., only a smaller portion of the dataset belonged to prey taxa (638,231 reads, 7975 prey reads per sample). Whether the discarded reads were assigned to contaminant, symbiont or consumer sources was not reported, but we find it likely that the majority was sourced from the consumer DNA. Likewise, a study of *Pseudocalanus* spp. copepods found 28,000 prey sequences in 46 samples (Cleary et al., 2016). PNA‐PCR was used also here to block amplification of the consumer, but as the authors did not report the initial read counts, the success rate of blocking is not known.

### 4.2 What influences prey sequence recovery?

There are many factors that can influence the recovery of prey sequences and thus the success of the brute force approach. The length of the target sequence is important, with small amplicons being favored due to a longer half‐life and hence an increased detectability (Kamenova et al., 2018). Whether the extracts are based upon recently ingested or heavily digested materials (e.g., feces) will also have an impact on recovery (Kamenova et al., 2018). For small invertebrates, whole‐body extraction is usually the only feasible alternative. With whole‐body samples, we find it probable that the consumer DNA has the greatest impact on prey sequence recovery as it competes for amplification and detection during PCR and sequencing. The severity of the overabundance problem is difficult to predict in advance, however, given that different consumers have variable genome sizes, cell numbers, and target gene copy numbers. Indeed, the ratio of the prey‐to‐consumer sequence may also vary from one season to the next, between different sexes, feeding, or life stages. The exact ratios acquired here may thus not be representative for studies of other invertebrates although the study design is otherwise identical. Nonetheless, we show that the problem of consumer DNA overabundance may be overcome by sequencing deeper.

With a great overabundance of putative consumer DNA (98%), our results underline the problem of dietary samples from small animals. In fact, over 3.8 billion of the sequence reads belonged to the top three abundant zOTUs, recruiting 1.6, 1.4, and 0.8 billion reads, respectively. We are confident that these represent the three sampled copepod species based on the abundance distribution and BLASTn searches of the NCBI nucleotide collection database (nt). The first zOTU dominated samples of *Oithona similis*, the second dominated samples from *Microcalanus* spp., and the third dominated samples from *Microsetella norvegica*. Other abundant maxillopod zOTUs with similar distribution patterns were also identified. These may be sequencing artifacts, individual or population level variants of the gene sequenced, or represent copepod prey. These zOTUs highlight a drawback to the approach, as the short 18S rRNA gene fragment (~100–110 bp) and PR2 database achieved high prey read numbers and good protist coverage, but sacrificed on taxonomic resolution of metazoans. Thus we were unable to confidently distinguish between copepod prey and consumers, and conservatively chose to discard all Maxillopoda sequence reads from our analyses. This could be problematic, given that many small invertebrates, also the copepods studied here—may feed on organisms with similar genetic signatures (e.g., copepods feeding on copepods). *Oithona nana* and *O. similis* may for instance feed on copepod nauplii (Lampitt, 1978; Nakamura & Turner, 1997), while *Microcalanus* spp. could have omnivore tendencies (Fortier et al., 2001; Schnack‐Schiel & Mizdalski, 1994). This limitation in the ability to identify feeding on closely related organisms can be mitigated somewhat by also sequencing the metazoan barcoding gene CO1 (cytochrome c oxidase subunit I), which provides better resolution of metazoan taxonomy.

It is however not just consumer DNA that is problematic as contaminants may further dilute samples, making finding enough prey data more difficult. In the datasets analyzed in this study, we observed more reads from the four extraction negatives sequenced for the pilot dataset (10.8 mill. reads) than in the combined 20 extraction negatives from the full dataset (9.3 mill. reads). This holds also at the level of real samples, given that the pilot dataset contains a higher contaminant percentage (1.6%) than the full dataset (1.1%). If we somehow “worked cleaner” during the preparation of the second sequencing run is not known, but we hypothesize that increased contamination could explain some of the observed gaps in prey recovery (0.4% in pilot vs. 1.2% in full dataset), since also contaminant sequences may compete for amplification and sequencing. A lot of non‐prey sequences would inevitably lead to a lower output of prey, and the more contaminated samples are the greater the problem. Moreover, contaminant sequences may have greater potential to compete during sequencing due to the low overall DNA concentrations from extracting individuals of small animals. Lowered elution volume or pooling several individuals per extraction may help toward this end. Future dietary studies may arguably increase the yield of prey sequences by paying attention to lab routines and making them as clean as possible. Further improvements could be made by employing additional extraction negatives, or through external bleaching of animals. The latter would have to be carefully tested, since the fiddly process of washing small consumers makes it difficult to give equal exposure to each sample, and because it remains unknown whether bleach may penetrate the small bodies to affect the dietary DNA.

Other possible influences on prey sequence recovery could be technical—for instance that deeper sequencing leads to a greater number of rare prey zOTUs surpassing a set cutoff value (we denoised each sample individually with a four reads cutoff), or related to the biology of the consumer. In addition to sequencing depth, sampling at an additional location and two more seasons in March and April–May, may have increased the number of unique prey zOTUs identified in the full dataset. Several Arctic copepod species may for example enter diapause prior to the polar night. A dormant state ensues, where energy spent on motility, reproduction, and feeding is drastically lowered (Conover, 1988). Because internal wax ester storages are used for energy, feeding activities are no longer required. Consequently, some copepod species may be expected to contain little or no prey DNA during certain periods of the year. The copepods studied here are not expected to enter diapause, but may to some extent reduce metabolic rates during winter (Conover & Huntley, 1991).

### 4.3 Advantages of the brute force method

We argue that for metabarcoding of dietary samples, the brute force method is superior to blocking primers by being simpler, cheaper, faster, and offering a less biased result. It is simpler because, theoretically, the trophic interactions of any animal may be sampled and studied regardless of whether it is well known to science or just recently discovered. With a blocking‐primer approach, the mandatory first step would be to have the targeted barcode of the consumer sequenced (unless it already exists). With sequence in hand, a site must be found that is unique and targets the consumer only. Site selection is difficult, however, because the blocking primer must be designed based on the sequences of both consumers (should match blocking primer perfectly) and putative prey (should be different enough to not bind the blocking primer). Ideally, one should also test the blocking primers against putative prey to make sure that the mismatches hinder hybridization. Services like TestProbe have been used to test blocking primers against broad databases like Silva (e.g., Ray et al., 2016), but it would be naive to expect that all putative prey sequences are available, especially for little‐known organisms sampled from frontier environments. Exactly how many nucleotide mismatches should be demanded, and at what point the blocking primers start affecting the amplification of other relevant sequences remains unknown. Piñol et al. (2015) found that amplification of nontarget DNA was blocked even when the number of mismatches were 4 and 5 base pairs. Also, prey studies often utilize extra short sequences that sacrifice on taxonomic resolution to allow detection of prey from partly digested material. These factors make the blocking primer approach particularly problematic. Designing primers that solely block the consumer within a short stretch of DNA can be difficult, and there are no guarantees of finding out whether the study is doable before investments in sanger sequencing, blocking primer design, or both have been made.

### 4.4 Costs of sequencing versus blocking primers

It is not straightforward to directly compare the costs of designing and applying blocking primers to that of ordering greater depth from a sequencing provider. If we, for example, were to perform our 20 μL PCR reactions with 20 μM PNA (e.g., Cleary et al., 2016, 2017) and with 150 samples per consumer, we would require 60 nanomole of each PNA primer. Using the lowest price inquired from two leading PNA manufacturers for the 18‐mer Cal‐PNA‐block PNA as an example (Ray et al., 2016), the starting costs for the quantities required here would amount to approximately $3900 for the three consumers. Moreover, we argue that the potential for upscaling sequencing depth is already great—and will continue to improve, as sequencing technology becomes ever more sophisticated and cost‐effective. Due to high demand and a growing customer base, sequencing providers are also forced to compete among themselves for customers, often leading to offers of discounts or even help with post‐sequencing computation. Meanwhile, PNA primer design and manufacture remains a time‐consuming and complicated chemical procedure that is less amenable to change or development.

## 5 CONCLUSIONS

With a small 18S rRNA gene fragment, we have shown that sufficient prey DNA may be acquired. The short read‐length coupled with a low resolution of metazoans, did however not allow for the identification of prey that are genetically highly similar to their consumer. We therefore call for further work to enable brute force metabarcoding with markers that better identify metazoans (e.g., CO1). Our results nevertheless show that brute force sequencing can serve as a simple and efficient approach for prey metabarcoding of consumers that are less known to science or difficult to dissect. Exactly how many extra reads one should ask for to offset the losses to consumer sequencing depends on multiple often unknown factors like copy number variation, life cycle, and seasonal parameters. We therefore suggest sequencing deeper than what is strictly required based on an arbitrary ratio (e.g., with expectancy of 99% consumer reads), as this approach is more cost‐efficient than resequencing samples with low coverage.

## AUTHOR CONTRIBUTIONS

**Snorre Flo:** Conceptualization (equal); data curation (lead); formal analysis (lead); investigation (lead); methodology (equal); resources (equal); software (lead); visualization (lead); writing – original draft (lead); writing – review and editing (equal). **Anna Vader:** Conceptualization (equal); funding acquisition (equal); methodology (equal); project administration (equal); resources (equal); supervision (equal); writing – review and editing (equal). **Kim Præbel:** Conceptualization (equal); funding acquisition (equal); methodology (equal); project administration (equal); resources (equal); supervision (equal); writing – review and editing (equal).

## CONFLICT OF INTEREST STATEMENT

The authors have no conflicts of interest to declare.

## CODE AVAILABILITY STATEMENT

Code for bioinformatics is openly available on github (https://github.com/snflo/bruteforce).

## Supporting information


Appendix S1.

## Data Availability

Raw data from Pilot and Full sequencing are available via the NIRD Research Data Archive in Flo (2023a, 2023b).
