# Supplementary material for: Brute force prey metabarcoding to explore the diets of small invertebrates
Source: Ecol Evol. 2024 May 6;14(5):e11369. doi: 10.1002/ece3.11369 (PMC11070772; doi:10.1002/ece3.11369)
Supplement: Supplementary file 1 — Appendix S1. [file ECE3-14-e11369-s001.docx]

Brute_force_Ecol_Evol_Supporting_Information_final

Supporting Tables

Table S1 – Primers and oligos used in this study.

| **Name** | **Size (bp)** | **Action** | **Sequence** | **Reference** |
| --- | --- | --- | --- | --- |
| 18S_allshortsF | ~100-110 | 18S V7 sequencing primers | 5’-TTTGTCTGSTTAATTSCG-3’ | (Guardiola et al., 2015) |
| 18S_allshortsR |  |  | 5’-TCACAGACCTGTTATTGC-3’ |  |
| NEXTflex^TM^ PCR-Free Barcoded Adapter |  | Indexed library adapter | AATGATACGGCGACCACCGAGATCTACACTCTTTCCCTACACGACGCTCTTCCGATCT  GATCGGAAGAGCACACGTCTGAACTCCAGTCACXXXXXX^1^ATCTCGTATGCCGTCTTCTGCTTG |  |
| Adapter 2 | 6 | Index library 1 | 5’-TGACCA-3’ |  |
| Adapter 3 | 6 | Index library 2 | 5’-ACAGTG-3’ |  |
| Adapter 4 | 6 | Index library 3 | 5’-GCCAAT-3’ |  |
| Adapter 5 | 6 | Index library 4 | 5’-CAGATC-3’ |  |
| Adapter 6 | 6 | Index library 5 | 5’-CTTGTA-3’ |  |

Guardiola, M., Uriz, M. J., Taberlet, P., Coissac, E., Wangensteen, O. S., & Turon, X. (2015). Deep-sea, deep-sequencing: Metabarcoding extracellular DNA from sediments of marine canyons. *PLoS ONE*, *10*(10), e0139633. https://doi.org/10.1371/journal.pone.0139633

Table S2 – Overview of copepods collected from four seasons and three stations and the positions of those (Latitude, Longitude). The maximum sampling depth to surface (Sd; m), bottom depth (Bd; m), and whether sea-ice was present (Yes/No) are presented for each net-sampling event. The numbers of biological replicates of each copepod picked and whose diet was assessed are shown with species acronyms (*O. similis*; Os, *Microcalanus* spp.; Mp, *M. norvegica*; Mn).

| \| Season \| Station \| Lat (°N) \| Lon (°E) \| Date \| Sd (m) \| Bd (m) \| Sea-ice \| Os \| Mp \| Mn \| \| --- \| --- \| --- \| --- \| --- \| --- \| --- \| --- \| --- \| --- \| --- \| \| Mar \| Shelf S \| 75.9999 \| 31.2196 \| 05.03.21 \| 300 \| 324 \| No \| 14 \| 14 \| 14 \| \| Shelf N \| 79.7662 \| 33.8264 \| 09.03.21 \| 320 \| 340 \| Yes \| 14 \| 14 \| 7 \| \| Basin \| 81.9989 \| 29.8381 \| 18.03.21 \| 1000 \| 3334 \| Yes \| 14 \| 14 \| NA \| \| Apr-May \| Shelf S \| 76.0000 \| 31.2202 \| 30.04.21 \| 300 \| 326 \| No \| 14 \| 13 \| 13 \| \| Shelf N \| 79.7438 \| 33.9800 \| 04.05.21 \| 320 \| 337 \| Yes \| 14 \| 14 \| 6 \| \| Basin \| 82.1422 \| 29.1633 \| 13.05.21 \| 1000 \| 3494 \| Yes \| 14 \| 14 \| NA \| \| Aug \| Shelf S \| 76.0000 \| 31.2200 \| 08.08.19 \| 300 \| 321 \| No \| 14 \| 14 \| 14 \| \| Shelf N \| 79.7211 \| 34.3182 \| 12.08.19 \| 330 \| 341 \| No \| 14 \| 14 \| 14 \| \| Basin \| 81.8291 \| 28.8017 \| 21.08.19 \| 1000 \| 2993 \| Yes \| 14 \| 14 \| 7 \| \| Dec \| Shelf S \| 76.0870 \| 31.0010 \| 13.12.19 \| 300 \| 333 \| No \| 14 \| 14 \| 14 \| \| Shelf N \| 79.7700 \| 34.0520 \| 08.12.19 \| 300 \| 326 \| Yes \| 14 \| 14 \| 13 \| \| Basin \| 82.1610 \| 28.1540 \| 04.12.19 \| 1000 \| 3660 \| Yes \| 14 \| 14 \| NA \| |
| --- | --- | --- | --- | --- | --- | --- | --- | --- | --- | --- | --- | --- | --- | --- | --- | --- | --- | --- | --- | --- | --- | --- | --- | --- | --- | --- | --- | --- | --- | --- | --- | --- | --- | --- | --- | --- | --- | --- | --- | --- | --- | --- | --- | --- | --- | --- | --- | --- | --- | --- | --- | --- | --- | --- | --- | --- | --- | --- | --- | --- | --- | --- | --- | --- | --- | --- | --- | --- | --- | --- | --- | --- | --- | --- | --- | --- | --- | --- | --- | --- | --- | --- | --- | --- | --- | --- | --- | --- | --- | --- | --- | --- | --- | --- | --- | --- | --- | --- | --- | --- | --- | --- | --- | --- | --- | --- | --- | --- | --- | --- | --- | --- | --- | --- | --- | --- | --- | --- | --- | --- | --- | --- | --- | --- | --- | --- | --- | --- | --- | --- | --- | --- | --- | --- | --- |

Table S3 – Summary of statistical tests used to assess differences in prey composition depending on season, station or species sampled (groups) for the three datasets (“Pilot”, “Pilot full”, and “Full”). Additional tests were performed on datasets agglomerated at Class-level taxonomy (not shown in manuscript). Bray-Curtis and Jaccard dissimilarity metrics (d_metric) were computed from relative abundance (relab) and presence-absence (pa) based tables, respectively. PERMANOVA statistics are reported with the *F*-statistic (*F*_perm), *p*-value (*p*_perm) and significance (* = *p* <0.05, ** = *p* <0.01, *** = *p* <0.001). Group dispersions are shown with *p*-values of the Betadisper test (*p*_betadisp) and identified dispersion (disp = homogenous or heterogenous). Fig_2 points to the relevant NMDS from the manuscript.

| **Dataset** | **taxlvl** | **data** | **d_metric** | **group** | **F_perm** | ***p*_perm** | **sig** | ***p*_betadisp** | **disp** | **Fig_2** |
| --- | --- | --- | --- | --- | --- | --- | --- | --- | --- | --- |
| Pilot | zOTU | relab | bray | season | 5.6 | 0.0001 | *** | 0.307 | homogenous | a |
| Pilot | zOTU | relab | bray | station | 12.6 | 0.0001 | *** | 0.449 | homogenous | a |
| Pilot | zOTU | relab | bray | species | 2.2 | 0.0028 | ** | 0.022 | heterogenous | a |
| Pilot full | zOTU | relab | bray | season | 5.2 | 0.0002 | *** | 0.002 | heterogenous | c |
| Pilot full | zOTU | relab | bray | station | 16.0 | 0.0001 | *** | 0.001 | heterogenous | c |
| Pilot full | zOTU | relab | bray | species | 1.7 | 0.0326 | * | 0.743 | homogenous | c |
| Full | zOTU | relab | bray | season | 24.5 | 0.0001 | *** | 0.052 | homogenous | e |
| Full | zOTU | relab | bray | station | 39.9 | 0.0001 | *** | 0.001 | heterogenous | e |
| Full | zOTU | relab | bray | species | 3.5 | 0.0001 | *** | 0.514 | homogenous | e |
| Pilot | zOTU | pa | jacc | season | 3.0 | 0.0001 | *** | 0.001 | heterogenous | b |
| Pilot | zOTU | pa | jacc | station | 2.7 | 0.0003 | *** | 0.099 | homogenous | b |
| Pilot | zOTU | pa | jacc | species | 2.3 | 0.0002 | *** | 0.215 | homogenous | b |
| Pilot full | zOTU | pa | jacc | season | 4.6 | 0.0001 | *** | 0.84 | homogenous | d |
| Pilot full | zOTU | pa | jacc | station | 4.1 | 0.0001 | *** | 0.298 | homogenous | d |
| Pilot full | zOTU | pa | jacc | species | 1.7 | 0.0001 | *** | 0.648 | homogenous | d |
| Full | zOTU | pa | jacc | season | 18.0 | 0.0001 | *** | 0.001 | heterogenous | f |
| Full | zOTU | pa | jacc | station | 7.3 | 0.0001 | *** | 0.001 | heterogenous | f |
| Full | zOTU | pa | jacc | species | 3.2 | 0.0001 | *** | 0.001 | heterogenous | f |
| Pilot | class | relab | bray | season | 6.3 | 0.0001 | *** | 0.291 | homogenous | NA |
| Pilot | class | relab | bray | station | 11.3 | 0.0001 | *** | 0.422 | homogenous | NA |
| Pilot | class | relab | bray | species | 2.3 | 0.0058 | ** | 0.06 | homogenous | NA |
| Pilot full | class | relab | bray | season | 10.1 | 0.0001 | *** | 0.039 | homogenous | NA |
| Pilot full | class | relab | bray | station | 10.5 | 0.0001 | *** | 0.001 | heterogenous | NA |
| Pilot full | class | relab | bray | species | 2.0 | 0.0228 | * | 0.332 | homogenous | NA |
| Full | class | relab | bray | season | 32.1 | 0.0001 | *** | 0.392 | homogenous | NA |
| Full | class | relab | bray | station | 22.6 | 0.0001 | *** | 0.001 | heterogenous | NA |
| Full | class | relab | bray | species | 3.3 | 0.0001 | *** | 0.154 | homogenous | NA |
| Pilot | class | pa | jacc | season | 2.7 | 0.0142 | * | 0.975 | homogenous | NA |
| Pilot | class | pa | jacc | station | 2.5 | 0.0216 | * | 0.873 | homogenous | NA |
| Pilot | class | pa | jacc | species | 2.5 | 0.0049 | ** | 0.74 | homogenous | NA |
| Pilot full | class | pa | jacc | season | 13.7 | 0.0001 | *** | 0.211 | homogenous | NA |
| Pilot full | class | pa | jacc | station | 9.0 | 0.0001 | *** | 0.073 | homogenous | NA |
| Pilot full | class | pa | jacc | species | 2.7 | 0.0025 | ** | 0.589 | homogenous | NA |
| Full | class | pa | jacc | season | 47.7 | 0.0001 | *** | 0.001 | heterogenous | NA |
| Full | class | pa | jacc | station | 6.2 | 0.0001 | *** | 0.194 | homogenous | NA |
| Full | class | pa | jacc | species | 6.0 | 0.0001 | *** | 0.002 | heterogenous | NA |

Table S4 – Mean relative abundance of prey taxa across all real samples from the full dataset (N = 437), and across each studied predator (*Microsetella norvegica*; Mn, *Microcalanus* spp.: Mp, *Oithona similis*; Os). Input zOTUs were filtered sample-wise by relative abundance (retaining zOTUs > 0.1% of sample).

| Supergroup | Division | Class | Global | Mn | Mp | Os |
| --- | --- | --- | --- | --- | --- | --- |
| Alveolata | Ciliophora | Cyclotrichium_like | 0.1 | 0.3 | 0.0 | 0.0 |
|  |  | Spirotrichea | 2.2 | 0.3 | 3.8 | 2.1 |
|  | Dinoflagellata | Dinophyceae | 6.1 | 4.3 | 5.4 | 8.0 |
|  |  | Dinophyta_X | 4.4 | 1.6 | 5.7 | 5.4 |
| Amoebozoa | Breviatea | Breviatea_X | 0.0 | 0.2 | 0.0 | 0.0 |
| Hacrobia | Haptophyta | Prymnesiophyceae | 0.2 | 0.4 | 0.1 | 0.2 |
| Opisthokonta | Choanoflagellida | Choanoflagellatea | 0.0 | 0.0 | 0.0 | 0.1 |
|  | Fungi | Ascomycota | 4.2 | 4.2 | 5.8 | 2.6 |
|  |  | Basidiomycota | 2.4 | 3.5 | 1.7 | 2.1 |
|  | Mesomycetozoa | Ichthyosporea | 0.0 | 0.0 | 0.1 | 0.0 |
|  | Metazoa | Annelida | 0.3 | 0.2 | 0.5 | 0.1 |
|  |  | Chaetognatha | 49.2 | 49.8 | 48.8 | 49.3 |
|  |  | Echinodermata | 0.4 | 0.3 | 0.6 | 0.2 |
|  |  | Nematoda | 0.0 | 0.0 | 0.1 | 0.0 |
|  |  | Nemertea | 0.1 | 0.0 | 0.1 | 0.0 |
|  |  | Rotifera | 1.8 | 1.6 | 2.5 | 1.3 |
|  |  | Urochordata | 8.0 | 11.2 | 6.2 | 7.5 |
|  | Opisthokonta_X | Opisthokonta_XX | 1.3 | 1.5 | 1.4 | 1.0 |
| Rhizaria | Cercozoa | Endomyxa-Ascetosporea | 0.0 | 0.0 | 0.0 | 0.1 |
|  |  | Filosa-Imbricatea | 0.1 | 0.5 | 0.0 | 0.0 |
|  |  | Filosa-Thecofilosea | 0.1 | 0.0 | 0.2 | 0.0 |
|  |  | Phaeodarea | 0.1 | 0.0 | 0.1 | 0.2 |
|  | Radiolaria | Acantharea | 1.1 | 1.3 | 1.2 | 0.9 |
|  |  | Polycystinea | 0.0 | 0.0 | 0.1 | 0.0 |
| Stramenopiles | Ochrophyta | Bacillariophyta | 16.1 | 16.3 | 14.3 | 17.7 |
|  |  | Chrysophyceae | 0.3 | 0.2 | 0.4 | 0.4 |
|  |  | Dictyochophyceae | 0.1 | 0.0 | 0.2 | 0.0 |
|  |  | Pelagophyceae | 0.1 | 0.1 | 0.0 | 0.1 |
|  | Opalozoa | MAST-3 | 0.1 | 0.3 | 0.0 | 0.1 |
|  | Pseudofungi | MAST-1 | 0.1 | 0.2 | 0.1 | 0.1 |
|  | Sagenista | Labyrinthulomycetes | 0.8 | 1.7 | 0.4 | 0.5 |

Supporting Figures


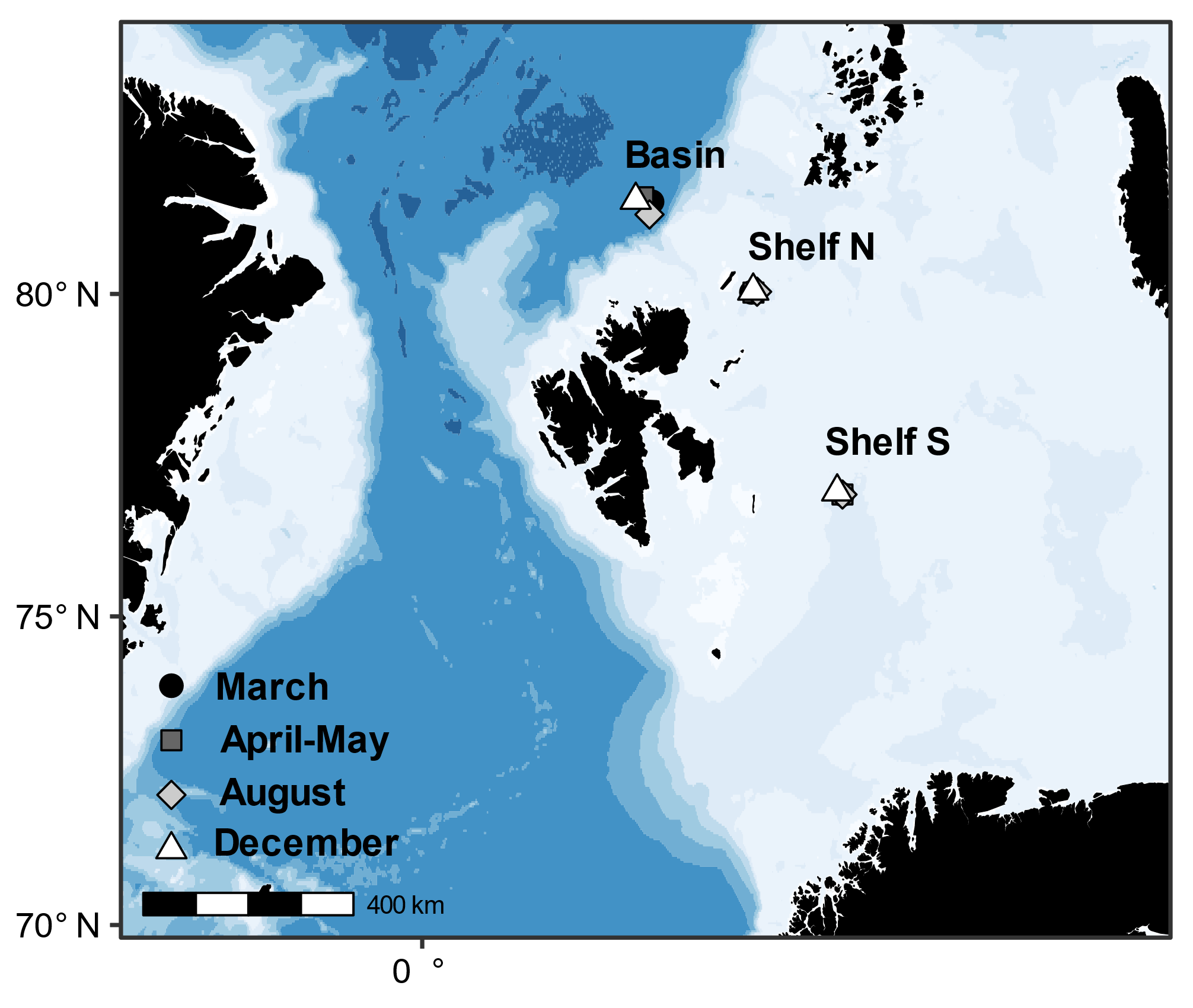


Figure S1 – Map of sampling stations located in the Barents Sea (Shelf S and Shelf N) and Nansen Basin (Basin). The stations were sampled on all four seasonal cruises as notified by different shapes and colors.
